# RELATIONSHIP BETWEEN SOCIAL ISOLATION AND EXPECTATION FOR PERMANENT LIVING IN A MARGINALIZED COMMUNITY IN JAPAN

**DOI:** 10.1093/geroni/igad104.2900

**Published:** 2023-12-21

**Authors:** Yuichi Watanabe

**Affiliations:** Musashino University, Nishitokyo, Tokyo, Japan

## Abstract

Social isolation is a predominant issue for the well-being of older adults in Japan. In marginalized communities, there is a high aging ratio of over 50%, and many older adults live alone. If they did not have connections in the community, there would be huge problems, not just loneliness but also the lack of necessities of daily life. This fact could threaten their permanent living. Therefore, This research examines the relationship between social isolation and permanent living expectation. Data were gathered by surveying a marginalized community every two years from 2015 to 2019 (N=435). Logistic regression analysis was conducted. For measuring social isolation, the questionnaire includes two items as independent valuables; “How many contacts do you have with community residents in a week?”, “How many contacts do you have with someone living out of this community in a week?.” An item measured permanent living expectation as a dependant valuable; “Would you like to be living here permanently when you live alone?” Gender, age, economic status, health status, and living alone were also included in the analysis. The results show that the number of contacts with community residents significantly predicted the permanent living expectation(P<.00, odds ratio=1.099). Health status is also significantly associated with a permanent living expectation. Findings suggest that social isolation in a community is a risk for giving up a permanent living. It could be social exclusion. Our effort to make mutual connections and contacts among the community residents could ease the risk of social exclusion.

